# Rapid Detection of Apixaban by a ROTEM-Based Approach and Reversibility with Andexanet Alfa or DOAC-Stop

**DOI:** 10.1055/s-0042-1751072

**Published:** 2022-08-29

**Authors:** Viktor Taune, Mika Skeppholm, Anna Ågren, Agneta Wikman, Andreas Hillarp, Håkan Wallén

**Affiliations:** 1 Department of Clinical Sciences, Danderyd Hospital, Division of Cardiovascular Medicine, Karolinska Institutet, Stockholm, Sweden; 2 Division of Clinical Immunology and Transfusion Medicine, Department of Laboratory Medicine, Karolinska University Hospital, Stockholm, Sweden; Department of Laboratory Medicine, Karolinska Institutet, Stockholm, Sweden; 3 Department of Translational Medicine, Clinical Chemistry Malmö, Lund University, Malmö, Sweden; 4 Department of Medical Biochemistry, Oslo University Hospital, Oslo, Norway

**Keywords:** atrial fibrillation, apixaban, drug monitoring, point-of-care test, thromboelastometry

## Abstract

**Background** A rapid test to detect apixaban treatment would be useful in acute situations such as major bleeding, urgent surgery, or in acute thrombosis.

**Objective** This article aims to study if the viscoelastic test rotational thromboelastometry (ROTEM) can rapidly detect apixaban in whole blood using modified triggers based on factor Xa (FXa) or Russell viper venom (RVV).

**Method** ROTEM clotting time (CT) was measured in samples from 40 patients on apixaban treatment, and in vitro in samples spiked with apixaban (20–500 ng/mL). Commercially available trigger Ex-tem was compared with modified triggers based on FXa or RVV. Reversibility of apixaban in the samples was studied; CT was measured with and without addition of DOAC-Stop or andexanet alfa, respectively, and the difference in CT was calculated (CT _diff_ ).

**Results** Using FXa as trigger, we detected apixaban concentrations at 20 ng/mL and above with 100% sensitivity and 100% specificity in patient samples and in vitro. Corresponding data for Ex-tem were 92% sensitivity and 100% specificity in patients, and 94% sensitivity and 100% specificity in vitro, and for RVV 97% sensitivity and 94% specificity in patients, and 97% sensitivity and 100% specificity in vitro, respectively. CT _diff_ data were similar. Patient sample data were obtained within 20 minutes from sampling.

**Conclusion** Apixaban at low therapeutic concentrations was detected within 20 minutes, and with high sensitivity and specificity. A trigger based on FXa outperformed the commercial trigger Ex-tem and a trigger based on RVV. ROTEM with a FXa-based trigger is a promising method to detect apixaban bedside in acute settings.

## Introduction

Apixaban is one of the direct oral anticoagulants (DOACs) rapidly replacing warfarin for treatment in atrial fibrillation (AF) and venous thromboembolism. 1 2 In contrast to warfarin, all DOACs have been approved without the need for routine monitoring. However, in situations such as major bleeding, acute thrombosis, or need for urgent invasive procedures, the value of determining the anticoagulant activity has been increasingly recognized. 3 4 5 6 As apixaban is by now one of the most prescribed and best-selling medications worldwide, 7 these situations should be increasingly common. In the pivotal Aristotle trial, the annual rate of major bleeding was 2%, 8 9 10 but higher in patients with comorbidities such as chronic kidney disease. 11 In practice-based health care outside large randomized controlled trials complications are, however, usually more common. Thus, in a “real-world” study of a U.S. population, major bleedings on apixaban were almost 5% per year 12 and even higher, close to 8%, in a Korean population. 13 Furthermore, bleeding complications in apixaban-treated patients which require actions from health care may occur in up to one-third of treated patients per year, according to data from U.S. 12 On the other side, there are now effective antidotes approved 14 or under clinical evaluation 15 for the reversal of apixaban. Of note, andexanet alfa (AA) (coagulation factor Xa [FXa] [recombinant] inactivated-zhzo), a FXa decoy, has been approved for patients treated with a direct FXa inhibitor (apixaban or rivaroxaban) when reversal of anticoagulation is needed, due to life-threatening bleeding or uncontrolled bleeding. 14 A recent meta-analysis of reversal agents for severe bleeding associated with DOACs showed that the risk of death was significantly associated with failure to achieve effective hemostasis, 16 supporting the contention that oral anticoagulation in the bleeding patient should be rapidly assessed and reversed when needed.

Thus, in patients on DOACs suffering from major bleeding or conditions demanding acute surgery, urgent assessment of anticoagulant activity is required, but there are few sensitive point-of-care (POC) tests to guide clinical decision making in these acute situations. Such a test would not necessarily need to determine the exact concentration. It would probably suffice to determine if clinically relevant concentration of anticoagulant is present or not. Rapid and sensitive POC tests would also be desirable considering the cost of antidotes, as acute assessment could aid in the identification of patients who really would benefit from a specific reversal therapy. 17 18

Of note, common routine coagulation test results with prothrombin time-international normalized ratio (PT-INR) or activated partial thromboplastin time lack sensitivity to with certainty detect or quantify apixaban or other DOACs and should not be used in acute situations. 19 20 The general opinion is that the concentration of the drug should be determined, as this has been shown to be closely linked to effects. 21 22 The gold standard for determination of DOAC concentration is liquid chromatography with tandem mass spectrometry (LC-MS/MS), 23 24 25 26 but this method is time consuming and less frequently available as a routine method. Plasma-based DOAC-specific coagulation assays with calibrators are available for all registered DOACs—diluted thrombin time for dabigatran and anti-FXa assays for the FXa inhibitors—and these are the methods mainly used in the clinic today, although they may not be available 24/7 at all hospitals. 3 23 24 25 It has also recently been put forward that rapid POC tests with appropriate performance are needed. 6

ROTEM (rotational thromboelastometry) and TEG (thromboelastography) are viscoelastic whole blood tests that can be used to guide treatment in perioperative and trauma settings. 27 The use of whole blood not only provides information on global hemostasis, but also shortens turnaround time and allows the test to be used POC. We have previously studied the use of ROTEM to determine effects of dabigatran. 28 29 We found that sensitivity varied with the different commercial triggers available, but none was sensitive enough to reliably detect clinically relevant concentrations of dabigatran. 28 With a modified trigger based on thrombin, that is, the trigger which would “pinpoint” actions of the specific thrombin inhibitor dabigatran, we could, however, accurately discriminate between samples with and without dabigatran down to drug concentrations as low as 20 ng/mL. 29 It should be emphasized that this concentration range is at the lowest measurable range for most plasma-based DOAC-specific coagulation assays, and from a clinical perspective associated with a significant increase in ischemic stroke, 30 thus detecting undertreatment and identifying patients free from anticoagulating effects of dabigatran. In addition, we compared the difference in clotting time (CT) between samples before and after addition of the dabigatran antidote idarucizumab. Since CT abnormalities from other causes than those mediated by dabigatran would not be reversed by this highly specific antidote, the CT difference could reveal the contribution of dabigatran in each sample and its reversibility.

The aim of the present study was to evaluate if ROTEM could be used to rapidly measure the effects of apixaban, and if the method could be improved by using modified triggers based on FXa or Russell viper venom (RVV), 31 that is, triggering compounds that exert procoagulation through the mechanism whereby apixaban inhibit coagulation and therefore sensitive to the presence of the drug. We also studied reversibility of apixaban with AA, or with DOAC-Stop (DS) which is a charcoal-based agent that has been shown to efficiently inhibit effects of DOACs present in samples intended for coagulation testing. 32 The turnaround time to obtain test results with the ROTEM-based approach was compared with test results from the local clinical laboratory at our hospital.

## Methods

### Patient and Donor Samples

The study was conducted in accordance with the Declaration of Helsinki and approved by the Ethical Review Board in Stockholm, Sweden (2012/1232–31; 2020–00924). Blood samples were collected from 40 patients (15 women and 25 men) on apixaban treatment admitted to the Department of Cardiology, Danderyd Hospital, Stockholm, Sweden, during the period September 30 to December 15, 2020. Indications for apixaban treatment were AF ( *n* = 39) and venous thromboembolism ( *n* = 1). Patients were treated according to summary product characteristics with apixaban 5 mg twice a day (32 patients) or 2.5 mg twice a day (8 patients). The patients had a median age of 74 years (range 55–85) and a median CHADS-VASc score 33 of 3 (range 0–6). The reasons for hospitalization were AF ( *n* = 25), heart failure ( *n* = 9), acute coronary syndrome ( *n* = 3), ventricular tachycardia ( *n* = 1), bleeding ( *n* = 1), and benign chest pain ( *n* = 1). Healthy donors were recruited through advertisement at the hospital and samples were collected during the period May 13 to December 15, 2020. Blood was collected by venipuncture in 3.2% citrate tubes (4.5 mL; Becton Dickinson, New Jersey, United States). All samples were analyzed using ROTEM as described below. Patient samples were analyzed for apixaban concentration using LC-MS/MS at the Department of Clinical Pharmacology at Karolinska University Hospital, Stockholm, Sweden. 24 In five patients, samples were also sent for emergency analysis of apixaban concentration using a chromogenic anti-FXa assay (STA Liquid Anti-FXa; Diagnostica Stago, Asnieres sur Seine, France) 24 available as a 24/7 routine analysis at the Department of Clinical Chemistry at Karolinska University Hospital (site Danderyd Hospital), to compare time from sampling until obtained results between the ROTEM assay and the chromogenic anti-FXa assay. In this setting, samples were sent by tube for urgent analysis to the Department of Clinical Chemistry before walking 5 to 6 minutes through the hospital to the placement of the ROTEM machine and then performing the ROTEM assays.

### Design of In Vitro Experiments

Blood from 14 healthy donors was incubated with apixaban (purity > 98%; Cayman Chemical, Michigan, United States) at estimated plasma concentrations of 0, 20, 50, 100, 300, and 500 ng/mL (assuming a hematocrit of 40%). The final concentration of solvent in the blood sample was 1%. Apixaban was acquired as a crystalline solid reconstituted with dimethyl sulfoxide to a stock solution and further diluted with acetonitrile water 1:1. Aliquots for each concentration were stored in –80°C before use.

### ROTEM Analyses and Triggers

ROTEM measurements were performed on a ROTEM delta apparatus (Tem Innovations GmbH, Munich, Germany). ROTEM analyses were performed at 37°C and in accordance with the manufacturer's recommendations. We used two different in-house triggers: one based on FXa and one based on RVV. In addition, we also used the commercially available trigger Ex-tem as this has been described to be the most sensitive trigger for measurements of apixaban treatment. 34 35

The FXa trigger consisted of bovine FXa (220 IU/mg; CoaChrom Diagnostica, Maria Enzerdorf, Austria) and phospholipids (phosphatidylcholine 42 mol%, phosphatidylserine 28 mol%, and sphingomyelin 30 mol%; Rossix, Mölndal, Sweden), the latter at a final whole blood concentration of 2 μM. The RVV trigger consisted of RVV (Diagnostic Reagents, Thame, United Kingdom) and phospholipids at a final whole blood concentration of 2 μM. FXa (delivered as a lyophilized powder) was reconstituted and diluted with distilled water and stored in aliquots at –80°C before use. RVV (acquired as a lyophilized powder) was stored at 2 to 8°C and reconstituted with distilled water and further diluted with tris buffer (50 mM Trizma base and 0.1 M NaCl in distilled water, adjusted to pH 7.4 with HCl) before each day's experiments. Phospholipids was delivered as an emulsion of phospholipids in tris buffer that was stored at 2 to 8°C and further diluted with tris buffer before each day's experiments. The trigger solution volume used was 20 μL and the whole blood volume in the test cup was 300 μL. All samples were recalcified with the commercially available calcium solution Star-tem (20 μL) before analysis.

ROTEM analyses were performed before and after incubation with the specific FXa inhibitor antidote AA (Alexion AstraZeneca Rare Disease, Massachusetts, United States) or DS (Haematex Research, Hornsby, Australia), respectively. One minitab of DS was added to 4.5 mL of whole blood. In clinical studies and package insert, AA is administered as a high dose (800 mg bolus followed by 8 mg/min continuous infusion during 120 minutes) or low dose (400 mg bolus followed by 4 mg/min continuous infusion during 120 minutes) depending on the size and timing of the last dose of DOAC. 14 We evaluated reversibility of samples with AA using a concentration equivalent to the maximum concentration of the high dose in samples from healthy volunteers (0.16 mg/mL) 36 as well as a high “supratherapeutic” concentration (0.64 mg/mL). Both reversal agents were incubated with each sample for at least 10 minutes before analysis. AA was not available to us when the study started. Therefore, fewer samples were analyzed in the presence of AA.

Based on results from previous studies, the only ROTEM variable registered was CT. 28 29 34 35 37 38 The difference in CT between samples with and without DS was evaluated as a separate variable, CT difference (CT _diff_ ), to assess the apixaban-specific effect and the potential reversibility of the drug effect. 29 Note that CT _diff_ has been calculated with DS as the reversal agent. The AA analyses have not been used in a similar manner due to fewer performed analyses (see above).

### Statistics

Sample sizes were calculated based on experiences from a similar study conducted by our group. 29 Analyses of receiver operating characteristics (ROCs) were used to evaluate CT and CT _diff_ with maximum speciﬁcity and sensitivity for discrimination between samples with and without apixaban. A *t* -test was used to compare the time until test results for the ROTEM assay and anti-FXa assay. When comparing three or more groups of results we used analysis of variance with post hoc testing (Tukey's or Dunnett's). For nonparametric data we used Kruskal–Wallis or Friedman with post hoc testing (Dunn's). Statistical analyses and figures were made using GraphPad Prism version 7 (GraphPad Software, Inc., California, United States).

### Data Sharing Statement

For original data, please contact viktor.taune@ki.se.

## Results

### Dose–Response Experiments

Dose–response curves were established to determine suitable concentrations of the FXa- and RVV-based triggers ( Supplementary Fig. S1 ). Based on these experiments we chose to use 0.05 IU/mL FXa and 1,500 ng/mL RVV as final trigger concentrations in the whole blood samples.

### ROTEM Experiments with Apixaban In Vitro

#### Effects of Apixaban

**Fig. 1:**
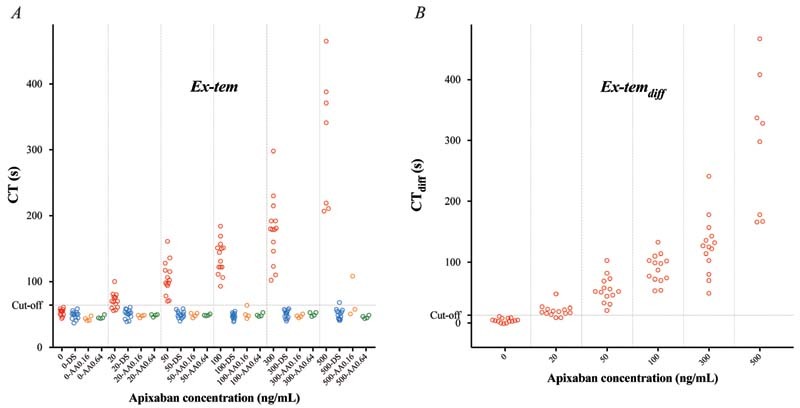
Rotational thromboelastometry (ROTEM) analyses using the Ex-tem trigger – variables clotting time (CT) ( **A** ) and CT _diff_ ( **B** ; the difference in CT without vs. with DOAC-Stop [DS]) – performed on blood samples from 14 healthy controls incubated with apixaban at estimated plasma concentrations 0, 20, 50, 100, 300, and 500 ng/mL (red). Samples were analyzed in the absence or presence of DOAC-Stop (DS; blue) and andexanet alfa (AA; performed in samples from 4 out of 14 donors) – the latter in two different concentrations (0.16 [orange] and 0.64 [green] mg/mL). Cutoff line represents optimal receiver operating characteristics (ROCs) for discriminating between samples with apixaban concentration above or below 20 ng/mL.

ROTEM analyses were performed on blood samples from 14 healthy individuals incubated with apixaban (20–500 ng/mL). CT was concentration-dependently prolonged by apixaban with a significant prolongation already at the lowest concentration tested (20 ng/mL), and with all three triggers ( *p* < 0.01 for all; Figs. 1 2 3A ; Supplementary Table S1 ). CT _diff_ 28 (difference between samples with and without DS) also increased significantly with all three triggers already at apixaban 20 ng/mL ( *p* < 0.01 for all; Figs. 1 2 3B ; Supplementary Table S1 ).

**Fig. 2:**
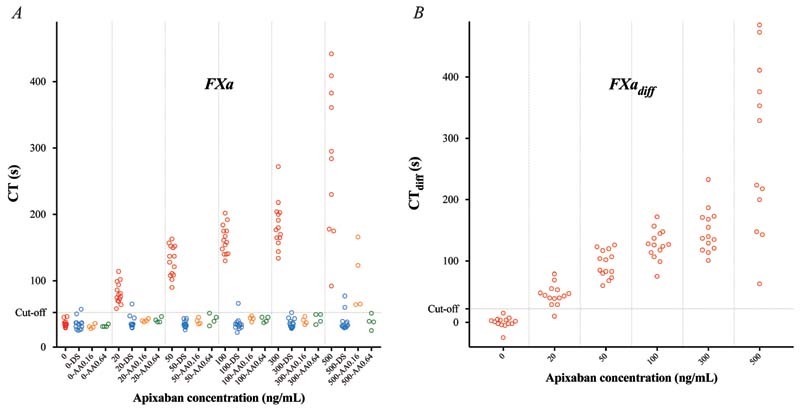
Rotational thromboelastometry (ROTEM) analyses using the factor Xa (FXa) trigger – variables clotting time (CT) ( **A** ) and CT _diff_ ( **B** ; the difference in CT without vs. with DOAC-Stop [DS]) – performed on blood samples from 14 healthy controls incubated with apixaban at estimated plasma concentrations 0, 20, 50, 100, 300, and 500 ng/mL (red). Samples were analyzed in the absence or presence of DOAC-Stop (DS; blue) and andexanet alfa (AA; performed in samples from 4 out of 14 donors) – the latter in two different concentrations (0.16 [orange] and 0.64 [green] mg/mL). Cutoff line represents optimal receiver operating characteristics (ROCs) for discriminating between samples with apixaban concentration above or below 20 ng/mL.

**Fig. 3:**
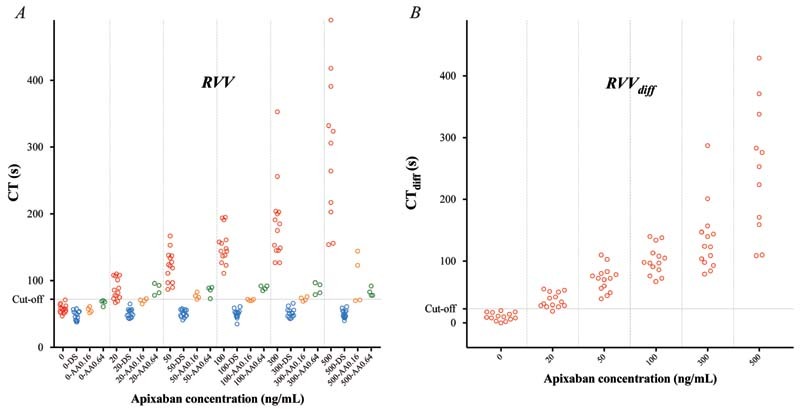
Rotational thromboelastometry (ROTEM) analyses using the Russell viper venom (RVV) trigger – variables clotting time (CT) ( **A** ) and CT _diff_ ( **B** ; the difference in CT without vs. with DOAC-Stop [DS]) – performed on blood samples from 14 healthy controls incubated with apixaban at estimated plasma concentrations 0, 20, 50, 100, 300, and 500 ng/mL (red). Samples were analyzed in the absence or presence of DOAC-Stop (DS; blue) and andexanet alfa (AA; performed in samples from 4 out of 14 donors) – the latter in two different concentrations (0.16 [orange] and 0.64 [green] mg/mL). Cutoff line represents optimal receiver operating characteristics (ROCs) for discriminating between samples with apixaban concentration above or below 20 ng/mL.

#### Receiver Operating Characteristics

ROCs were calculated to assess the ability of each trigger to detect apixaban concentrations *above* 20 ng/mL ( Table 1 ). This cutoff represents a concentration at the lowest limit of the range of concentrations seen in patients on apixaban treatment. 24 39 It is also the lowest concentration that can be measured with most plasma-based specific anti-Xa assays. 40 All three triggers detected apixaban with high sensitivity and specificity, but FXa-triggered CT outperformed the other two triggers (100% sensitivity and 100% specificity).

**Table 1:** Optimal receiver operating characteristics (ROCs) for discriminating between samples with apixaban concentration above or below 20 ng/mL in both the in vitro material and patient material

		Cutoff (s)	Sensitivity (%)	Specificity (%)
In vitro data	Ex-tem CT	64	94 (86–98)	100 (77–100)
	FXa CT	52	100 (95–100)	100 (77–100)
	RVV CT	72	97 (90–100)	100 (77–100)
	Ex-tem CT _diff_	13	97 (90–100)	100 (77–100)
	FXa CT _diff_	22	99 (92–100)	100 (77–100)
	RVV CT _diff_	23	99 (93–100)	100 (77–100)
Patient data	Ex-tem CT	62	92 (79–98)	100 (79–100)
	FXa CT	57	100 (91–100)	100 (79–100)
	RVV CT	89	97 (86–100)	94 (70–100)
	Ex-tem CT _diff_	9	87 (73–96)	88 (62–98)
	FXa CT _diff_	21	97 (87–100)	100 (79–100)
	RVV CT _diff_	22	97 (87–100)	94 (70–100)

Abbreviations: CT, clotting time; FXa, factor Xa; RVV, Russell viper venom.

Note: Sensitivity and specificity presented as mean with a 95% confidence interval (CI).

#### Reversibility of Apixaban Effects

All samples were analyzed with and without added DS. In samples with added DS, CT was normalized using the FXa trigger ( Fig. 2A ; Supplementary Table S1 ). Of note, RVV- and Ex-tem-triggered CT was slightly shorter than control sample in the presence of DS, both with and without added apixaban ( *p* < 0.01 for both triggers; Figs. 1 and 3A ; Supplementary Table S1 ).

In samples from 4 out of 14 donors, ROTEM analyses were performed in the absence or presence of AA in two different concentrations (0.16 and 0.64 mg/mL). In Ex-tem and FXa-triggered samples, AA normalized CT in all samples except for in those with the highest apixaban concentration (500 ng/mL) to which the lower AA concentration (0.16 mg/mL) was added ( *p* < 0.01 for both triggers; Figs. 1 and 2A ; Supplementary Table S1 ). In RVV-triggered samples, addition of AA shortened CT in samples with apixaban close to the CT seen for the control sample with added AA, but full reversal was not seen ( *p* < 0.01; Fig. 3A ; Supplementary Table S1 ). In fact, RVV-triggered CT was longer when spiked with 0.64 mg/mL AA, except for in samples containing the highest concentration of apixaban (500 ng/mL). In samples without apixaban, AA at 0.64 mg/mL caused a slight prolongation of RVV-triggered CT ( *p* < 0.05), suggesting a small inhibiting effect of AA on RVV-induced coagulation.

### ROTEM Experiments with Samples from Patients on Apixaban Therapy

#### ROTEM Measurements in Patient Samples

**Fig. 4:**
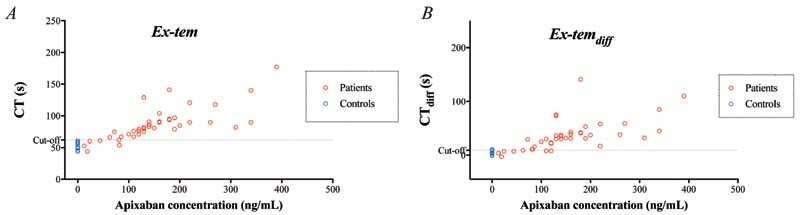
Rotational thromboelastometry (ROTEM) analyses using the Ex-tem trigger – variables clotting time (CT) ( **A** ) and CT _diff_ ( **B** ; the difference in CT without vs. with DOAC-Stop [DS]) – performed on blood samples from 40 patients on apixaban treatment. Variables presented in relation to apixaban concentration measured by liquid chromatography with tandem mass spectrometry (LC-MS/MS). Cutoff line represents optimal receiver operating characteristics (ROCs) for discriminating between samples with apixaban concentration above or below 20 ng/mL.

CT and CT _diff_ were longer in samples from patients on apixaban treatment than in control samples from healthy individuals without treatment, using all three triggers ( *p* < 0.01; Figs. 4 5 6 ; Supplementary Table S2 ). With a cutoff at 20 ng/mL and performing ROC, we detected apixaban with very high sensitivity and specificity with all three triggers but, as in the in vitro experiments, FXa-triggered CT stood out among the triggers, with 100% sensitivity and 100% specificity ( Table 1 ).

**Fig. 5:**
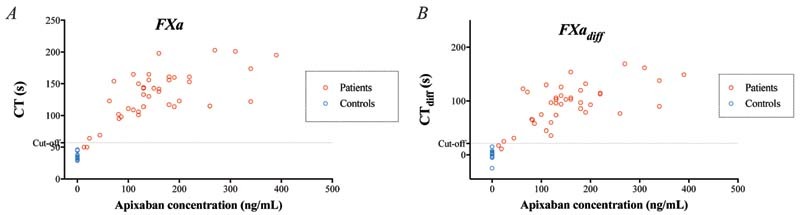
Rotational thromboelastometry (ROTEM) analyses using the factor Xa (FXa) trigger – variables clotting time (CT) ( **A** ) and CT _diff_ ( **B** ; the difference in CT without vs. with DOAC-Stop [DS]) – performed on blood samples from 40 patients on apixaban treatment. Variables presented in relation to apixaban concentration measured by liquid chromatography with tandem mass spectrometry (LC-MS/MS). Cutoff line represents optimal receiver operating characteristics (ROCs) for discriminating between samples with apixaban concentration above or below 20 ng/mL.

**Fig. 6:**
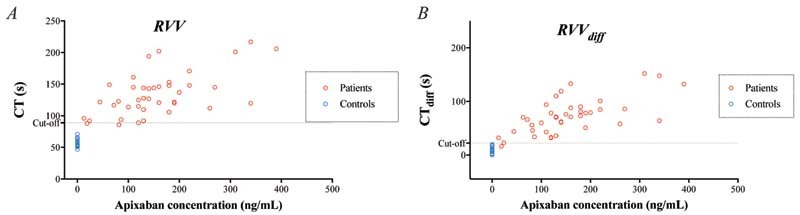
Rotational thromboelastometry (ROTEM) analyses using the Russell viper venom (RVV) trigger – variables clotting time (CT) ( **A** ) and CT _diff_ ( **B** ; the difference in CT without vs. with DOAC-Stop [DS]) – performed on blood samples from 40 patients on apixaban treatment. Variables presented in relation to apixaban concentration measured by liquid chromatography with tandem mass spectrometry (LC-MS/MS). Cutoff line represents optimal receiver operating characteristics (ROCs) for discriminating between samples with apixaban concentration above or below 20 ng/mL.

#### Reversibility of Apixaban Effects

All samples were analyzed with and without added DS. In the presence of DS, CT was normalized in all three triggers ( Supplementary Fig. S2 and Supplementary Table S2 ). In samples from 10 out of 40 patients, ROTEM analyses were performed in the absence or presence of AA in two different concentrations (0.16 and 0.64 mg/mL). In the presence of AA, CT was normalized in all samples triggered by Ex-tem, but less so with the FXa trigger ( *p* < 0.01), and even less with the RVV trigger ( *p* < 0.01). Of note, RVV-triggered CT tended to be concentration-dependently prolonged by AA in both patient and control samples ( Supplementary Fig S2 ; Supplementary Table S2 ).

#### Turnaround Time for Data on Apixaban in Patient Samples

Results from the ROTEM assay were available within a considerably shorter time frame (19 ± 1 minutes from venipuncture) compared with the emergency analysis of apixaban using a chromogenic anti-FXa assay performed at the clinical chemistry laboratory at our hospital (65 ± 5 minutes from venipuncture) ( *n* = 5; *p* < 0.01).

## Discussion

In this study we demonstrate that the ROTEM assay can be used to rapidly and with high sensitivity and specificity detect apixaban in whole blood samples from patients on treatment with this drug. The patient data were supported by results from in vitro experiments. We found that a trigger based on FXa outperformed a trigger based on RVV, and the commercially available trigger Ex-tem aimed to be used in the ROTEM assay. Further, we analyzed samples with and without the specific FXa-inhibitor antidote AA, or with and without the DOAC removal agent DS, thereby revealing reversibility in each sample. We found that the Ex-tem trigger, as well as a FXa-based and an RVV-based trigger, could detect apixaban in concentrations as low as 20 ng/mL in whole blood samples from patients on apixaban treatment. The clinical data were further supported by in vitro studies in samples from healthy donors spiked with apixaban. Of note, 20 ng/mL represents a plasma concentration in the very low therapeutic range 24 39 and is at the lowest measurable range for most plasma-based specific anti-Xa assays. 40 Our method is thus sensitive and should be potentially useful also to detect undertreatment with apixaban, which may be valuable, for example, in patients on apixaban suffering from acute ischemic stroke where thrombolysis is indicated. Furthermore, results from the ROTEM assay were available within 20 minutes of venipuncture, which was considerably faster than the results obtained with a chromogenic anti-FXa assay performed as a routine emergency analysis at the hospital laboratory. A POC test that can rapidly and accurately detect anticoagulant effects of DOACs would certainly be highly valuable in the acute setting in patients on DOAC treatment who are bleeding or are in need for urgent surgery. 3 4 5 6 21 Moreover, in clinical situations where the use of expensive and potent DOAC antidotes are considered, 17 18 a test which can help to rapidly identify patients who would benefit from reversal of anticoagulation, would be valuable and increase cost-effectiveness of antidotes.

The present study is not large enough to detect statistically significant differences in performance between the triggers with respect to ROC analyses. However, the FXa-based trigger stood out with 100% sensitivity and 100% specificity, and the RVV-based trigger seemed more sensitive than Ex-tem. Indeed, FXa-based triggers have been studied previously. In one study, an FXa-based trigger could accurately detect apixaban treatment in healthy volunteers, although with another viscoelastic instrument than ROTEM (the TEG6s). However, the study was small and there were very few samples with low apixaban concentrations in the study. 41 Other studies have used ROTEM to measure the effect of apixaban, and found that of the commercial triggers provided in the assay, the most sensitive was Ex-tem, but even with this trigger it was not possible to accurately detect therapeutic concentrations of the drug. 34 35 37 38 There are also studies on ROTEM which have shown that diluted Ex-tem or a low amount of tissue factor increased sensitivity to apixaban treatment, but these triggers did not appear accurate enough for clinical use. 42 43 In a study of another viscoelastic test, ClotPro, an RVV trigger detected apixaban concentrations above 50 ng/mL in trauma patients with 80% sensitivity and 88% specificity. 44 It is reasonable to assume that triggers which activate coagulation in a FX-dependent manner, such as FXa or RVV, should more easily detect effects of apixaban compared with a tissue factor-based trigger like Ex-tem. On the other hand, when it comes to assessment of more global aspects of hemostasis, which may be needed in a patient with severe bleeding, the Ex-tem trigger may be more suitable. It should, however, be very easy to perform additional ROTEM measurements on Ex-tem-induced coagulation in the acute situation.

We evaluated reversibility of samples with AA using a concentration equivalent to the maximal concentration obtained in studies of healthy volunteers (0.16 mg/mL), 36 and we also studied a high “supratherapeutic” concentration (0.64 mg/mL) corresponding to a concentration around four times higher than the plasma peak attained in the study in healthy controls. Using the FXa and the Ex-tem trigger, AA in a concentration of 0.16 mg/mL completely reversed apixaban in a concentration range between 20 and 300 ng/mL, whereas the effects of the highest concentration of apixaban tested, that is, 500 ng/mL (double upper range of therapeutic concentrations) was not completely reversed. The higher concentration of AA (0.64 mg/mL) was required to fully reverse the effect of 500 ng/mL apixaban. These results may seem reasonable given the relatively small molar excess of AA at 0.16 mg/mL (estimated to 3.6 × ; 1,350 vs. 370 pmol) compared with at 0.64 mg/mL (14 × ; 5,400 vs. 370 pmol). Further studies are needed to investigate what concentrations of AA are needed to reverse very high or “toxic” levels of apixaban.

In the reversibility experiments, we also found that DS and AA (at a high concentration), had some effects on CT apart from the inhibition of apixaban. We found that CT was slightly shorter than normal in the presence of DS when analyzed with the RVV and Ex-tem triggers, but not with the FXa trigger. DS appears to have a procoagulant effect apart from its inhibiting effect on apixaban. This agrees with a previous study in which DS was reported to attenuate thrombin generation though extraction of tissue factor pathway inhibitor. 45 Further, when DS is used in plasma-based coagulation tests the activated charcoal is centrifuged from the sample before analysis. In these experiments the activated charcoal remained in the whole blood samples. This could also have affected the results. In our experiments, AA also had some influence on coagulation. When using RVV as trigger, AA prolonged CT in a concentration-dependent manner in the absence of apixaban. Furthermore, in apixaban-treated samples triggered by RVV, CT was shortened but not fully normalized by AA, and was somewhat longer with the higher concentration of AA. Together these observations suggest that AA interferes with the procoagulant effect of RVV. Similar findings have been reported for AA in a RVV assay. The mechanism was suggested to be competition between AA and FX in the binding of RVV. 46 47 RVV thus seems less well suited to be used as trigger, and possible batch-to-batch variation as well as species variations in venom composition, also makes RVV less appealing. 48

The composition and concentrations of added phospholipids have been shown to influence results obtained with RVV in the dilute RVV time test. 49 In our study, we used phospholipids from Rossix in a known composition, and at a concentration previously used by us to study dabigatran. 29 The composition and concentration of phospholipids in the Ex-tem trigger is, however, not disclosed and we cannot exclude that this influenced the results.

The difference between samples with and without DS was evaluated as a separate variable, the CT _diff_ . The rationale behind CT _diff_ would be to reveal the contribution of apixaban in the test and its reversibility with respect to drug-induced CT prolongation. With this approach some of the interindividual variability in CT response to apixaban could be reduced, including possible coagulation abnormalities in acutely ill patients, apart from those dependent on apixaban. However, in this study in clinically stable patients, data from the CT _diff_ experiments did not add any incremental value.

## Strengths and Limitations

This is a proof-of-concept study with a limited number of observations. A strength of the study is that it compares the two most promising triggers to detect apixaban treatment with a commercial trigger in blood samples from patients as well as in vitro. It should be noted that the sensitivity and specificity for detecting apixaban above 20 ng/mL of the different triggers only apply to the patient and control population investigated in this study. A proper validation of the method requires a larger study, preferably in patients with apixaban-related bleeding complications or in patients on apixaban in need for urgent surgery. The assays would also likely be sensitive to other anticoagulants to some degree and differing between different types of anticoagulant treatment is not tested in this study. However, it is often known with which anticoagulant the patient is treated and the question is rather how anticoagulated the patient is with the specific treatment. For example, PT-INR is not specific for warfarin treatment but is still used to guide treatment in warfarin-treated patients. Another strength is the comparison of time until results between ROTEM and the chromogenic anti-FXa assay. However, it remains to be tested if the same speed and quality of ROTEM experiments could be maintained when used as a true POC test in larger scale in a clinical setting. Further, specific triggers for DOACs would have to be produced and stored, but as all experiments were performed using commercially available products and instruments, replication studies should be easy to perform. Other limitations are that not all samples were analyzed in the presence of AA and that in vitro concentrations were not confirmed by measurement of apixaban concentration.

## Conclusion

ROTEM with a modified trigger based on FXa detected apixaban down to concentrations in the low therapeutic range, and more accurately than the commercial trigger Ex-tem or a trigger based on RVV. Results from the ROTEM assay were available within 20 minutes, which was much faster than when analyzed with a plasma-based specific coagulation assay at the local clinical chemistry laboratory. DS and AA can be used to reverse the effects of apixaban in the ROTEM assay, but AA is probably not well suited for RVV assays due to interference with procoagulant effect of RVV. DS has a slight procoagulant effect when used in whole blood that should be considered but is also cheaper and probably easier to store and use. We believe that our method is valuable in the clinical handling of patients on apixaban treatment with acute severe complications.

## Highlights

- A rapid test to detect apixaban treatment would be useful in acute situations.
- Viscoelastic test ROTEM was used to measure effects of apixaban in whole blood.
- Apixaban at concentrations as low as 20 ng/mL was accurately detected.
- FXa-based trigger outperformed commercial trigger Ex-tem and RVV-based trigger.
