# Supplementary material for: Rapid Detection of Apixaban by a ROTEM-Based Approach and Reversibility with Andexanet Alfa or DOAC-Stop
Source: TH Open. 2022 Aug 29;6(3):e238–47. doi: 10.1055/s-0042-1751072 (PMC9423941; doi:10.1055/s-0042-1751072)
Supplement: Supplementary file 1 — Supplementary Material [file 10-1055-s-0042-1751072-s220021.pdf]

Supplementary Table S1 ROTEM analyses with Ex-tem, FXa, and RVV reagents performed on blood samples from 14 healthy controls incubated with apixaban at estimated plasma concentrations 0, 20, 50, 100, 300, and 500 ng/mL

Apixaban concentration (ng/mL)	0	20	50	100	300	500
Ex-tem CT	55 (44–61)	70 (56–100)	100 (70–161)	138 (93–184)	180 (102–298)	493 (207–1425)
Ex-tem CT - DS	51 (37–59)	52 (39–61)	50 (40–59)	48 (39–55)	52 (40–59)	50 (41–68)
Ex-tem CT - AA 0.16	43 (41–48)	49 (45–49)	50 (45–52)	49 (44–64)	48 (45–51)	58 (51–108)
Ex-tem CT - AA 0.64	45 (44–50)	50 (46–50)	49 (48–51)	49 (47–53)	51 (47–53)	46 (44–49)
Ex-tem CT_{diff}	4 (–1 to 11)	19 (9–48)	52 (21–103)	93 (53–133)	126 (49–241)	438 (166–1376)
FXa CT	35 (29–46)	78 (58–114)	133 (90–163)	160 (130–202)	180 (134–272)	372 (92–1202)
FXa CT - DS	35 (25–57)	34 (29–65)	35 (26–43)	34 (22–66)	34 (28–52)	33 (29–77)
FXa - AA 0.16	31 (28–36)	40 (38–43)	38 (35–45)	44 (38–47)	39 (34–46)	94 (64–166)
FXa CT - AA 0.64	31 (31–35)	40 (38–46)	42 (32–51)	42 (37–45)	44 (34–49)	39 (25–51)
FXa CT_{diff}	2 (–25 to 15)	44 (10–79)	94 (60–126)	127 (75–172)	139 (101–233)	341 (63–1164)
RVV CT	58 (47–71)	84 (67–110)	123 (87–167)	146 (111–195)	180 (127–353)	328 (154–2156)
RVV CT - DS	48 (38–58)	50 (43–65)	51 (41–58)	51 (35–61)	51 (43–66)	49 (40–61)
RVV - AA 0.16	56 (52–61)	71 (65 (73)	76 (72–83)	71 (70–72)	73 (69–76)	97 (70–144)
RVV CT - AA 0.64	69 (61–70)	88 (78–96)	88 (73–90)	90 (85–92)	88 (79–97)	81 (78–92)
RVV CT_{diff}	10 (0–20)	33 (19–55)	72 (39–110)	98 (67–140)	124 (79–287)	280 (109–2101)

Abbreviations: AA, andexanet alfa; CT, clotting time; DS, DOAC-Stop; FXa, factor Xa; ROTEM, rotational thromboelastometry; RVV, Russell viper venom.
Note: Samples were analyzed in the absence or presence of DOAC-Stop (DS) and andexanet alfa (AA; performed in samples from 4 out of 14 donors) – the latter in two different concentrations (0.16 and 0.64 mg/mL). CT_{diff} is a variable that represents the difference in CT without vs. with DS in the same samples. Data presented as median and range.

Supplementary Table S2 ROTEM analyses with Ex-tem, FXa, and RVV reagents performed on blood samples from 40 patients on apixaban treatment

	Patients	Healthy controls
Apixaban concentration (ng/mL)	140 (13–390)	0 (0–0)
Ex-tem CT	82 (44–177)	55 (44–61)
Ex-tem CT - DS	52 (43–73)	51 (37–59)
Ex-tem CT - AA 0.16	52 (45–59)	43 (41–48)
Ex-tem CT - AA 0.64	58 (49–66)	45 (44–50)
Ex-tem CT_{diff}	31 (–3 to 141)	4 (–1 to 11)
FXa CT	136 (50–203)	35 (29–46)
FXa CT - DS	38 (28–70)	35 (25–57)
FXa - AA 0.16	46 (34–49)	31 (28–36)
FXa CT - AA 0.64	47 (43–53)	31 (31–35)
FXa CT_{diff}	97 (0–169)	2 (–25 to 15)
RVV CT	126 (86–217)	58 (47–71)
RVV CT - DS	64 (33–102)	48 (38–58)
RVV - AA 0.16	88 (77–108)	56 (52–61)
RVV CT - AA 0.64	109 (94–139)	69 (61–70)
RVV CT_{diff}	70 (0–152)	10 (0–20)

Abbreviations: AA, andexanet alfa; CT, clotting time; DS, DOAC-Stop; FXa, factor Xa; ROTEM, rotational thromboelastometry; RVV, Russell viper venom.
Note: Samples were analyzed in the absence or presence of DOAC-Stop (DS) and andexanet alfa (AA; performed in samples from 10 out of 40 patients) – the latter in two different concentrations (0.16 and 0.64 mg/mL). CT_{diff} is a variable that represents the difference in CT without vs. with DS in the same samples. Data presented as median and range.

 © 2022. The Author(s).

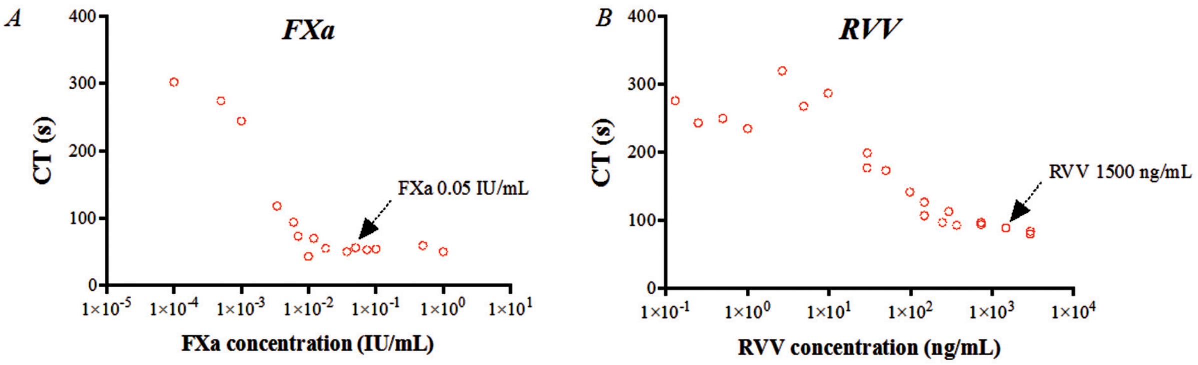

Supplementary Fig. S1 Initial experiments: whole blood rotational thromboelastometry (ROTEM) analyses on blood samples from a single donor using different concentrations of the factor Xa (FXa) (**A**) and Russell viper venom (RVV) (**B**) reagents to create dose–response curves. Arrows indicate the concentrations chosen for the remainder of experiments. At an apixaban concentration of 20 ng/mL, FXa at a concentration of 0.05 IU/mL corresponds to a 5× (15 vs. 3 pmol) molar excess of apixaban, giving a reasonable chance of detecting an anticoagulant effect.

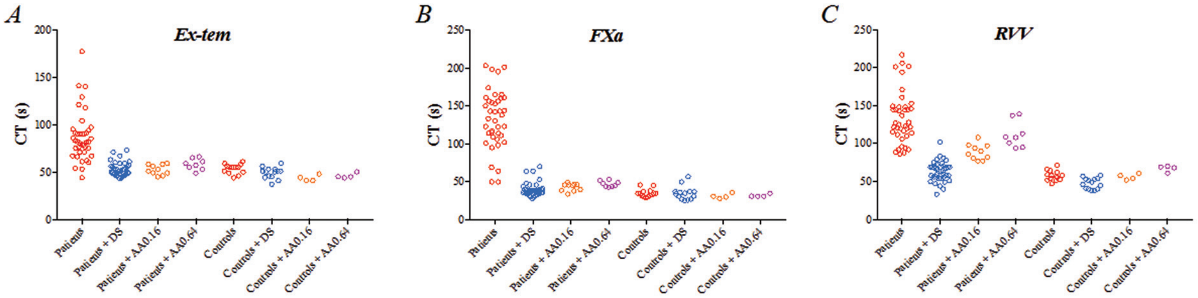

Supplementary Fig. S2 Whole blood rotational thromboelastometry (ROTEM) analyses – Ex-tem (**A**), factor Xa (FXa) (**B**), and Russell viper venom (RVV) (**C**) reagents – on blood samples from 40 patients on apixaban treatment (red) in comparison to 14 healthy controls (red). Samples were analyzed in the absence or presence of DOAC-Stop (DS; blue) and andexanet alfa (AA; performed in samples from 10 out of 40 patients) – the latter in two different concentrations (0.16 [orange] and 0.64 mg/mL [purple]).

 © 2022. The Author(s).
